# Tetanus Toxoid Vaccine Uptake and Associated Factors Among Reproductive Age Women in Mogadishu, Somalia: A Cross-Sectional Study

**DOI:** 10.1016/j.ijregi.2025.100804

**Published:** 2025-11-13

**Authors:** Amina Abukar Abdulle, Shafie Abdulkadir Hassan

**Affiliations:** 1 Department of Applied Research & Statistics, Center for Postgraduate Studies, Jamhuriya University of Science and Technology, Mogadishu, Somalia; 2 Faculty of Medicine and Health Sciences, Jamhuriya University of Science and Technology, Mogadishu, Somalia

**Keywords:** Tetanus toxoid, Vaccine uptake, Associated factors, Reproductive-age women, Somalia

## Abstract

**Objectives:**

Maternal and neonatal tetanus (MNT) remains a major public health concern in Somalia, where health infrastructure is still recovering. Despite the availability of a safe and effective tetanus toxoid (TT) vaccine, coverage remains low. This study aimed to identify factors influencing TT vaccine uptake among reproductive-age women in Mogadishu, Somalia.

**Methods:**

A community-based cross-sectional study was conducted from June to August 2025 in Mogadishu, Somalia. A two-stage cluster sampling technique combined with systematic random sampling was used to select 350 households. Data were collected using a structured, interviewer-administered questionnaire, and the primary outcome was adherence to at least two doses of the TT vaccine during pregnancy. Data were analyzed using bivariate and multivariable logistic regression to identify independent predictors of TT vaccine uptake.

**Results:**

A total of 350 women participated in the study. Coverage of the TT vaccine was low, with only 19.7% receiving two or more doses during their last pregnancy. Multivariate analysis showed that women aged 36-49 years were more likely to be protected compared to those aged 15-25 years (AOR = 6.62; 95% confidence interval [CI]: 1.74-25.21; *P* = 0.006). Planned pregnancy was associated with higher protection (AOR = 8.35; 95% CI: 3.25-21.45; *P* <0.001). Attendance at health centers for antenatal care (AOR = 0.35; 95% CI: 0.18-0.69; *P* = 0.002), visits by health extension workers (AOR = 8.13; 95% CI: 4.35-15.22; *P* <0.001), and good behavior of health workers (AOR = 3.48; 95% CI: 1.15-10.46; *P* = 0.027) were positively associated with TT protection. Women from households with a monthly income below 57 US$ were significantly less likely to be protected (AOR = 0.04; 95% CI: 0.01-0.22; *P* <0.001).

**Conclusions:**

TT vaccination coverage among women was low. Older age, planned pregnancy, antenatal care at health centers, visits by health extension workers, and good behavior of health workers were associated with higher protection, while low household income was linked to lower uptake. Strengthening targeted interventions is needed to improve TT coverage.

## Highlights

• Tetanus toxoid vaccine coverage in Mogadishu is low (19.7% received ≥2 doses).
• Older women and those with planned pregnancies were more protected.
• Visits by health extension workers greatly increased protection.
• Antenatal care attendance and respectful health worker behavior boosted uptake.
• Low-income women were much less likely to be protected.

## Introduction

Maternal and neonatal tetanus (MNT) is a fatal but preventable disease caused by the bacterium *Clostridium tetani* [1]. Globally, Maternal and Neonatal Tetanus persists as a public health challenge, particularly in regions with low immunization coverage and limited access to hygienic childbirth practices [[2], [3], [4]]. The World Health Organization (WHO) has targeted the global elimination of MNT, a goal contingent on achieving high coverage of the tetanus toxoid (TT) vaccine among women of childbearing age [5,6]. Crucially, the prevention of neonatal tetanus relies on the passive transfer of maternal antibodies to the fetus, a process highly dependent on the mother adhering to the recommended TT vaccination schedule during pregnancy [7]. Immunizing a pregnant woman with at least two doses of the TT vaccine can reduce neonatal tetanus mortality by up to 94% [5,8].

While significant progress has been made globally, Maternal and Neonatal Tetanus Elimination (MNTE) remains an unfinished agenda in several countries, including Somalia [9,10]. Decades of civil conflict have fragmented Somalia's health system, leading to some of the world's poorest health indicators [11,12]. A recent study on tetanus cases at a major hospital in Mogadishu found that 85.7% of admitted patients had never received a tetanus vaccination, underscoring the tragic consequences of low immunization coverage in the city [13]. However, general vaccination coverage does not capture the critical dimension for neonatal protection, which is adherence to the specific antenatal TT vaccination schedule. While prior immunization provides some maternal protection, only timely vaccination during pregnancy ensures that a sufficient concentration of antibodies is transferred to the fetus, providing passive immunity through the vulnerable neonatal period [14,15]. While research from neighboring Ethiopia has identified determinants of TT uptake such as education, antenatal care (ANC) attendance, and health worker visits, there is a profound gap in contemporary, context-specific evidence from Somalia [16]. Understanding the unique barriers and enablers in a post-conflict, urban setting like Mogadishu is essential for developing effective public health strategies.

This study aims to bridge this critical knowledge gap by assessing the level of adherence to the WHO recommended TT vaccination schedule during pregnancy and identifying its key determinants among women in Mogadishu. The findings are intended to provide an evidence base for policymakers and health organizations to design targeted interventions that improve not only overall vaccine coverage but, crucially, the timely completion of the antenatal TT vaccination series to advance progress toward MNT elimination in Somalia.

## Methods

### Study design and setting

A community-based cross-sectional study was conducted in Mogadishu, Somalia, between June and August 2025. The city’s health system comprises public, private, and NGO-run facilities,operating within a context of post-conflict reconstruction and ongoing security challenges.

### Study population and sampling

The study population comprised women who had delivered a child within the 12 months preceding the survey. A systematic random sampling technique was used to select participants from different districts of the city to ensure a representative sample.

### Sample size determination

The sample size was calculated using the formula for estimating a single proportion:

$$n=\frac{Z^{2}\cdot p\cdot \left(1-p\right)\cdot DEFF}{d^{2}\cdot \left(1-r\right)}$$

Where:

• Z = 1.96 for 95% confidence
• p = 0.17 (prevalence of TT vaccination, SDHS 2020)
• d = 0.05 (margin of error)
• DEFF = 1.5 (design effect for cluster sampling)
• r = 10% (anticipated non-response rate)

The calculated sample size was 326 participants, and after accounting for the 10% anticipated non-response, the final sample size was rounded to 350 participants to ensure feasibility and adequate representation.

### Sampling techniques

A community-based study was conducted in Mogadishu using a two-stage cluster sampling technique. Districts and divisions were randomly selected, households were chosen systematically, and one eligible woman per household was included.

### Data collection

Data were collected using a pre-tested, interviewer-administered questionnaire. The tool gathered information on several domains:

• **Sociodemographic characteristics:** Maternal age, marital status, maternal and husband's education level, maternal and husband's occupation, and average monthly household income.
• **Reproductive and health service factors:** Parity, ANC attendance and timing, place of delivery, interactions with health workers (respectfulness, behavior), perceived quality of service, and visits from a health extension worker (HEW). Information on visits by HEWs was obtained by asking participants whether they had been visited by an HEW during their most recent pregnancy.
• **TT vaccination status**: Uptake of the TT vaccine during the last pregnancy and number of doses.

### Operational definitions

• **Protected:** Women who received two or more doses of the TT vaccine, providing adequate protection against neonatal tetanus.
• **Not protected:** Women who received only one dose or none, indicating inadequate or no protection against neonatal tetanus.

### Statistical analysis

Data were analyzed using descriptive statistics to summarize participant characteristics. The primary outcome was the uptake of two or more doses of the TT vaccine. Bivariate analysis was conducted to determine the associations between independent variables and TT vaccine uptake. A multivariable logistic regression model was constructed to identify the independent predictors of vaccination, controlling for potential confounders. Adjusted odds ratios (AORs) with 95% confidence intervals (CIs) were calculated, and a *P*-value of <0.05 was considered statistically significant.

## Results

### Participant characteristics

A total of 350 women of reproductive age participated in the study. Most respondents (63.4%) were aged 26-35 years, and the majority were married (87.1%). Nearly half of the women (44.3%) were unable to read or write, while only 13.4% had attained a tertiary education. Husbands were generally better educated, with 32.6% having a tertiary-level education and 27.7% having no formal education. Most women were housewives (64.9%), and the majority of husbands were daily laborers (78.6%). Household income was low, with 35.1% of families earning less than 57 US$ per month, and about 54.9% of respondents had access to television or radio (Table 1).

**Table 1:** Sociodemographic characteristics of respondents.

Variables	Category	Frequency (N)	Percentage
**Maternal age**	15-25 years	79	22.6
	26-35 years	222	63.4
	36-49 years	49	14.0
**Marital status**	Married	305	87.1
	Divorced	32	9.1
	Widowed	13	3.7
**Maternal education**	Unable to write and read	155	44.3
	Primary (Grade 1-8)	70	20.0
	Secondary (Grade 9-12)	78	22.3
	Tertiary (Diploma or Degree)	47	13.4
**Maternal occupation**	Housewife	227	64.9
	Merchant	10	2.9
	Daily laborer	113	32.3
**Monthly household income ($)**	<57	123	35.1
	57-94	84	24.0
	94-141	39	11.1
	>141	104	29.7
**Husband education level**	No formal education	97	27.7
	Primary	90	25.7
	Secondary	49	14.0
	Tertiary	114	32.6
**Husband’s occupation**	Government employee	15	4.3
	Merchant	18	5.1
	Daily laborer	275	78.6
	Others (e.g., Private employee, Student)	42	12.0
**TV or radio**	Yes	192	54.9
	No	158	45.1

Regarding reproductive and maternal health, most women (60.9%) had 2-3 children, and 92.9% expressed a desire for future fertility. Approximately 62.6% reported their last pregnancy was planned. ANC follow-up was attended by 77.1% of women, with 71.1% having 2-3 visits and 23.1% attending four or more visits. The majority initiated ANC visits within the first trimester (75.4%), primarily at hospitals (78.9%), and most deliveries occurred in health institutions (75.7%) (Table 2).

**Table 2:** Reproductive and maternal health characteristics of respondents (N = 350).

Variable	Category	Frequency (n)	Percentage (%)
**Parity status**	1 time	70	20.0
	2-3 times	213	60.9
	4 or more times	67	19.1
**Future fertility intention**	Yes	325	92.9
	No	25	7.1
**Planned last pregnancy**	Yes	219	62.6
	No	131	37.4
**ANC follow-up**	Yes	270	77.1
	No	80	22.9
**Number of ANC visits**	1 visit	19	5.4
	2-3 visits	249	71.1
	4 or more visits	82	23.1
**Timing of first ANC visit**	<13 weeks	264	75.4
	13-28 weeks	58	16.6
	>28 weeks	6	1.7
	Don’t know	22	6.3
**Place of ANC visit**	Health center	74	21.1
	Hospital	276	78.9
**Place of delivery**	Home	85	24.3
	Health institution (Post, Center, Hospital, Clinic)	265	75.7

ANC, antenatal care.

In terms of health service access and quality, most respondents perceived health workers as respectful (84.3%) and well-behaved (90.0%), with 82.0% rating service quality as good. Trust in healthcare providers was reported by 76.3% of women. About two-thirds (65.7%) reached a health facility within 30 minutes, but only 34.0% received visits from health extension workers (Table 3).

**Table 3:** Health service-related factors among respondents (N = 350).

Variable	Category	Frequency (n)	Percentage (%)
Health worker respectfulness	Yes	295	84.3
	No	55	15.7
Quality of health service	Good	287	82.0
	Poor	63	18.0
Trust in health provider	Yes	267	76.3
	No	83	23.7
Behavior of health workers	Good	315	90.0
	Poor	35	10.0
Time to reach health facility	<30 minutes	230	65.7
	30 minutes-1 hour	73	20.9
	>1 hour	47	13.4
Health extension worker visit	Yes	119	34.0
	No	231	66.0

### TT vaccination uptake

**Figure 1:**
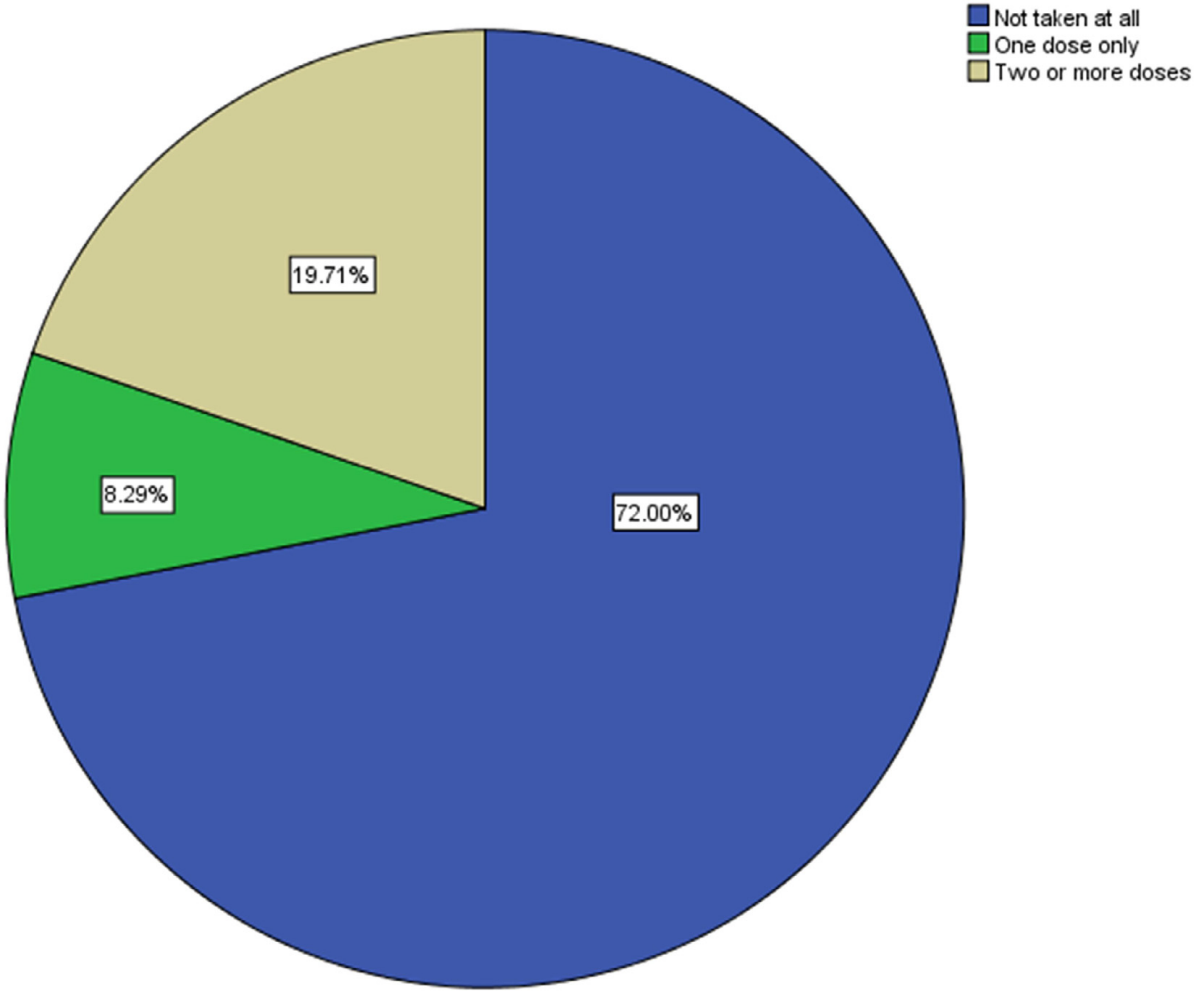
Distribution of tetanus toxoid vaccination status among reproductive age women.

TT vaccination coverage was low among the study population. The majority of women (72.0%) had not received any TT dose, 8.3% had received only one dose, and 19.7% had received two or more doses (Figure 1).

### Factors associated with TT vaccine uptake

Several sociodemographic, reproductive, and health service factors were associated with TT vaccination among reproductive-age women.

### Sociodemographic factors

Maternal age, occupation, household income, and husband’s education were significantly associated with TT protection. Women aged 36-49 years were more likely to be protected than those aged 15-25 years (AOR = 6.615; 95% CI: 1.736-25.210; *P* = 0.006). Housewives were less likely to be protected than daily laborers (AOR = 2.393; 95% CI: 1.131-5.063; *P* = 0.023). Women from households earning 57-94 US$ per month were more likely to be protected than those earning less than 57 US$ (AOR = 0.042; 95% CI: 0.008-0.215; *P* = 0.001). Higher husband education levels were also positively associated with TT protection (Table 4).

**Table 4:** Sociodemographic factors associated with tetanus toxoid uptake among women.

Variable	Category	Protected N (%)	Not protected N (%)	Crude OR (95% CI)	Adjusted OR (95% CI)	*P*-value
**Maternal age (years)**	15-25	27 (34.6)	52 (19.1)	1	1	
	26-35	47 (60.3)	175 (64.3)	1.933 (1.098-3.403)	2.079 (962-4.490)	0.063
	36-49	4 (5.1)	45 (16.5)	5.841 (1.900-17.963)	6.615 (1.736-25.210)	0.006
**Marital status**	Married	65 (83.3)	240 (88.2)	0.671 (0.145-3.105)	1.087 (144-8.227)	0.936
	Divorced	11 (14.1)	21 (7.7)	0.347 (0.065-1.851)	0.871 (111-6.848)	0.895
	Widowed	2 (2.6)	11 (4)	1	1	
**Maternal education**	illiterate	34 (43.6)	121 (44.5)	1.510 (0.726-3.139)	1.698 (458-6.296)	0.428
	Primary	11 (14.1)	59 (21.7%)	2.275 (0.928-5.582)	2.332 (697-7.799)	0.169
	Secondary	19 (24.4)	59 (21.7)	1.317 (0.585-2.965)	0.919 (321-2.631)	0.875
	Tertiary	14 (17.9)	33 (12.1)	1	1	1
**Maternal occupation**	Housewife	39 (50)	188 (69.1)	2.442 (1.451-4.112)	2.393 (1.131-5.063)	0.023
	Merchant	1 (1.3)	9 (3.3)	4.560 (0.557-37.332)	24.298 (1.914-308.497)	0.014
	Daily laborer	38 (48.7)	75 (27.6)	1	1	
**Monthly Household income ($)**	<57	14 (17.9)	109 (40.1)	1.854 (0.885-3.885)	0.279 (061-1.264)	0.098
	57-94	32 (41)	52 (19.1)	0.387 (0.201-0.746)	0.042 (008-215)	0.001
	94-141	12 (15.4)	27 (9.9)	0.536 (0.232-1.237)	0.139 (032-608)	0.009
	>141	20 (25.6)	84 (30.9)	1	1	1
**Husband education level**	No formal education	29 (37.2)	68 (25)	1	1	
	Primary	15 (19.2)	75 (27.6)	2.132 (1.054-4.313)	6.424 (1.813-22.766)	0.004
	Secondary	7 (9)	42 (15.4)	2.559 (1.029-6.361)	10.473 (2.431-45.117)	0.002
	Tertiary	27 (34.6)	87 (32)	1.374 (0.745-2.536)	4.798 (1.384-16.632)	0.013
**Husband’s occupation**	Government employee	2 (2.6)	13 (4.8)	2.600 (0.508-13.301)	0.748 (106-5.283)	0.771
	Merchant	5 (6.4)	13 (4.8%)	1.040 (0.304-3.557)	0.990 (197-4.974)	0.990
	Daily laborer	59 (75.6)	216 (79.4)	1.464 (0.707-3.035)	1.451 (472-4.461)	0.515
	Private sector employee	12 (15.4)	30 (11)	1	1	1
**TV/Radio exposure**	Yes	33 (42.3)	159 (58.5)	1	1	
	No	45 (57.7)	113 (41.5%)	1.919 (1.152-3.195)	0.808 (365-1.787)	0.598

CI, confidence interval; OR, odds ratio.

### Reproductive factors

Women whose last pregnancy was planned were more likely to be protected than those with unplanned pregnancies (AOR = 8.347; 95% CI: 3.248-21.454; *P* = 0.001). Receiving ANC at health centers was associated with higher protection compared to hospital ANC (AOR = 0.347; 95% CI: 0.175-0.687; *P* = 0.002). Other reproductive factors, including parity, fertility intention, ANC attendance, and number of ANC visits, were not significantly associated with TT protection (Table 5).

**Table 5:** Reproductive factors associated with tetanus toxoid uptake.

Variable	Category	Protected N (%)	Not protected N (%)	Crude OR (95% CI)	Adjusted OR (95% CI)	*P*-value
**Parity status**	1 time	13 (16.7)	57 (21)	0.861 (0.356-2.084)	0.650 (0.232-1.825)	0.414
	2-3 times	54 (69.2)	159 (58.5)	0.578 (0.283-1.184)	0.462 (0.203-1.049)	0.065
	≥4 times	11 (14.1)	56 (20.6)	1	1	—
**Future fertility intention**	Yes	75 (96.2)	250 (91.9)	2.200 (0.641-7.554)	0.403 (0.090-1.799)	0.234
	No	3 (3.8)	22 (8.1)	1	1	—
**Planned last pregnancy**	Yes	71 (91)	148 (54.4)	8.498 (3.772-19.147)	8.347 (3.248-21.454)	0.001
	No (Ref)	7 (9.0)	124 (45.6)	1	1	—
**ANC follow-up**	Yes	63 (80.8)	207 (76.1)	1.319 (0.704-2.472)	1.467 (0.728-2.957)	0.284
	No	15 (19.2)	65 (23.9)	1	1	—
**Number of ANC visits**	1 visit	2 (2.6)	17 (6.2)	3.517 (0.754-16.414)	2.658 (0.515-13.716)	0.243
	2-3 visits	52 (66.7)	197 (72.4)	1.568 (0.891-2.759)	1.145 (0.573-2.286)	0.701
	≥4 visits	24 (30.8)	58 (21.3)	1	1	—
**Timing of first ANC visit**	<13 weeks	55 (70.5)	231 (84.9)	0.840 (0.096-7.335)	0.618 (0.052-7.328)	0.703
	13-28 weeks	22 (28.2)	36 (13.2)	0.327 (0.036-2.988)	0.432 (0.035-5.369)	0.514
	>28 weeks	1 (1.3)	5 (1.8)	1	1	—
**Place of ANC visit**	Health center	32 (41.0	42 (15.4)	0.263 (0.150-0.459)	0.347 (0.175-0.687)	0.002
	Hospital	46 (59)	230 (84.6)	1	1	—
**Place of delivery**	Home	11 (14.1)	74 (27.2)	2.276 (1.140-4.544)	2.117 (0.979-4.580)	0.057
	Health institution (Post, Center, Hospital, Clinic)	67 (85.9)	198 (72.8)	1	1	—

ANC, antenatal care; CI, confidence interval; OR, odds ratio.

### Health service-related factors

Women who received visits from health extension workers were significantly more likely to be protected than those who did not (AOR = 8.134; 95% CI: 4.346-15.222; *P* = 0.001). Good behavior from health workers also increased protection compared to poor behavior (AOR = 3.475; 95% CI: 1.154-10.463; *P* = 0.027). Other service-related factors, such as respectfulness, perceived service quality, trust in providers, and travel time to the facility, were not significantly associated with TT protection (Table 6).

**Table 6:** Health service access and quality factors associated with tetanus toxoid uptake.

Variable	Category	Protected	Not protected	Crude OR (95% CI)	Adjusted OR (95% CI)	*P*-value
**Health worker respectfulness**	Yes	70 (89.7)	225 (82.7)	1.828 (0.824-4.052)	1.602 (0.659-3.890)	0.298
	No	8 (10.3)	47 (17.3)	1	1	—
**Quality of health service**	Good	71 (91)	216 (79.4)	0.380 (0.166-0.872)	0.646 (0.251-1.660)	0.364
	Poor	7 (9)	56 (20.6)	1	1	—
**Trust in health provider**	Yes	64 (82.1)	203 (74.6)	1.554 (0.820-2.945)	1.185 (0.554-2.532)	0.662
	No	14 (17.9)	69 (25.4)	1	1	—
**Behavior of health workers**	Good	72 (92.3)	243 (89.3)	0.698 (0.279-1.748)	3.475 (1.154-10.463)	0.027
	Poor	6 (7.7)	29 (10.7)	1	1	—
**Time to reach facility**	<30 minutes	57 (73.1)	173 (63.6)	0.207 (0.062-0.692)	0.282 (0.077-1.028)	0.055
	30 min-1 hour	18 (23.1)	55 (20.2)	0.208 (0.058-0.753)	0.359 (0.089-1.444)	0.149
	>1 hour	3 (3.8)	44 (16.2)	1	1	—
**Health extension worker visit**	Yes	55 (70.5)	208 (76.5)	7.772 (4.432-13.627)	8.134 (4.346-15.222)	0.001
	No (Ref)	23 (29.5)	64 (23.5)	1	1	—

CI, confidence interval; OR, odds ratio.

## Discussion

This study reveals a critically low TT vaccine coverage of 19.7% among reproductive-age women in Mogadishu, which is far below the WHO’s MNT elimination target of 80% coverage [17]. This highlights the vulnerability of mothers and newborns in Somalia and underscores the urgent need for targeted interventions.

Maternal age was significantly associated with TT protection in this study, with women aged 36–49 years being more likely to be protected than younger women. This finding is consistent with other studies from similar contexts, which also reported higher TT uptake among older mothers [18,19].

Maternal occupation was significantly associated with TT uptake: housewives were less likely to be protected compared to daily laborers, and women in merchant occupations had markedly higher odds. In contrast, a community-based study in Ethiopia found that government employees had significantly higher TT uptake than housewives, while merchants did not show a significant association [5].

Household income was positively associated with TT protection. Women from lower-income households (<57 US$ per month) were less likely to be protected, while those in moderate-income households (57–94 US$) had higher odds of being vaccinated. This pattern suggests that economic barriers may limit access to maternal immunization services. Similar findings have been reported in other low-resource settings, where lower household income was consistently linked to reduced TT uptake [18,20].

Husband’s education emerged as a strong predictor of TT protection, with higher education levels associated with increased vaccine uptake. This finding is similar to studies in Africa, where women with more educated spouses were significantly more likely to receive TT immunization [16,21]. This emphasizes the influential role of male partners in maternal health decisions and highlights the need to engage men in health promotion interventions.

Reproductive factors also played a significant role: women whose last pregnancy was planned were more likely to be protected. This may be because planned pregnancies often reflect greater preparedness, increased contact with ANC services, and heightened health-seeking behavior. A study in northern Ethiopia similarly found that women with a planned pregnancy were over six times more likely to receive the protective dose of the TT vaccine than those with unplanned pregnancies [22]. Attending ANC at health centers rather than hospitals was associated with higher TT protection, likely because health centers may offer more accessible, frequent, and community-based care, including vaccination during ANC. A similar finding was reported in Ethiopia, where women who accessed ANC from a health center had significantly higher TT uptake compared to those who visited hospitals [16].

Our finding that women who received home visits from health extension workers were significantly more likely to be protected underscores the critical role of community-based outreach. A similar study in Ethiopia found that mothers who had frequent household visits by HEWs were 1.29 times more likely to utilize maternal health services compared to those who did not [23]. This suggests that investing in and expanding community health worker programs is arguably the single most effective strategy for boosting maternal immunization in Somalia.

These findings collectively indicate that TT uptake is influenced by maternal and husband characteristics, reproductive intentions, the type of ANC facility attended, community-based outreach, and the quality of interactions with healthcare providers. Consequently, health promotion strategies in Mogadishu should not only target women but also actively engage male partners, strengthen and expand community health worker programs, and prioritize the delivery of respectful, high-quality care.

## Conclusion

TT vaccine coverage among reproductive-age women in Mogadishu remains critically low, leaving most mothers and newborns vulnerable to a preventable but potentially fatal disease. Vaccine uptake is influenced by maternal and husband characteristics, reproductive intentions, the type of ANC facility attended, community-based outreach, and the quality of interactions with healthcare providers. These findings highlight the need for public health strategies that go beyond general awareness campaigns to include targeted, family-centered interventions, active engagement of male partners, expansion of community health worker programs, and the delivery of respectful, high-quality care.

### Limitation of the study

The study is subject to limitations. As a cross-sectional study, it cannot establish causality. The reliance on self-reported vaccination status may introduce recall bias, particularly given that over half of the women lacked vaccination cards. Finally, the findings from urban Mogadishu may not be generalizable to rural or other regions of Somalia.

## Funding

This research received no specific grant from any funding agency in the public, commercial, or not-for-profit sectors.

## Ethical approval

Ethical approval for this study was granted by the Ethical Review Board of the National Institute of Health (NIH), Somalia (Reference No: NIH/IRB/50/JUNE/2025). The study was conducted in compliance with the Declaration of Helsinki. Informed consent was obtained from all participants prior to their inclusion in the study.

## Author contributions

AAA conducted the investigation, including the acquisition of data, contributed to the formal data analysis, and wrote the original draft of the manuscript. SAH conceptualized the study, developed the methodology, supervised the project, participated in the data analysis, and critically reviewed and edited the manuscript for important intellectual content. Both authors have read and approved the final version to be published and agree to be accountable for all aspects of the work.

## Declaration of competing interest

The authors have no competing interests to declare.

## Appendix Supplementary materials

Image, application 1
