# Supplementary material for: Tetanus Toxoid Vaccine Uptake and Associated Factors Among Reproductive Age Women in Mogadishu, Somalia: A Cross-Sectional Study
Source: IJID Reg. 2025 Nov 13;18:100804. doi: 10.1016/j.ijregi.2025.100804 (PMC12775988; doi:10.1016/j.ijregi.2025.100804)
Supplement: Supplementary file 1 [file mmc1.pdf]

Ref: NIH/IRB/50/JUNE/2025 **06/26/2025/Mogadishu**

Subject: **Study Authorization**

Dear Amina Abukar Abdulle, (Investigator),

Your Proposal **Tetanus Toxoid Vaccine Uptake and Associated Factors among Reproductive-age Women in Mogadishu, Somalia: A Cross Sectional Study,** upon expedited review of critical information about the proposed study, it was agreed that the proposed study does not cause more than the minimum risk. This is therefore to inform you that the Ethical Review Board of the National Institute of Health (NIH) - Somalia has granted approval for your study.

Note that your study approval number is **NIH/IRB/50/JUNE/2025**. Reference this number in any correspondences with the research directorate of NIH.

Continued approval is conditional upon your compliance with the following:

1. Permission must be sought from relevant authorities for this study.
2. Informed consent must be obtained from respective study respondents before commencement of any data collection procedures and must be documented.
3. Any significant changes in the approved study that have the potential to negatively affect the safety and welfare of the study respondents must be reported immediately to the research directorate of NIH.
4. A report should be sent to the research directorate of NIH within 90 days of completion of the study

IRB Decision: Approved

Dr Hussein Abukar Muhiadin **Mukhtar Bulale**

Director General, NIH Acting Research Director, NIH

National Institute of Health, Via Londra, Hamarweyne, Mogadishu - Somalia
Email: info@nih.gov.so/research@nih.gov.so, Tel: +252619555575

NIH
NATIONAL INSTITUTE OF HEALTH - SOMALIA

Federal Republic Of Somalia

nih.gov.so
Info@nih.gov.so

Ref: NIH/IRB/50/JUNE/2025 **06/26/2025/Mogadishu**

Subject: **Study Authorization**

Dear Amina Abukar Abdulle, (Investigator),

Your Proposal **Tetanus Toxoid Vaccine Uptake and Associated Factors among Reproductive-age Women in Mogadishu, Somalia: A Cross Sectional Study,** upon expedited review of critical information about the proposed study, it was agreed that the proposed study does not cause more than the minimum risk. This is therefore to inform you that the Ethical Review Board of the National Institute of Health (NIH) - Somalia has granted approval for your study.

Note that your study approval number is **NIH/IRB/50/JUNE/2025**. Reference this number in any correspondences with the research directorate of NIH.

Continued approval is conditional upon your compliance with the following:

1. Permission must be sought from relevant authorities for this study.
2. Informed consent must be obtained from respective study respondents before commencement of any data collection procedures and must be documented.
3. Any significant changes in the approved study that have the potential to negatively affect the safety and welfare of the study respondents must be reported immediately to the research directorate of NIH.
4. A report should be sent to the research directorate of NIH within 90 days of completion of the study

IRB Decision: Approved

Dr Hussein Abukar Muhiadin

Director General, NIH

Mukhtar Bulale

Acting Research Director, NIH
